# Application of *Swietenia macrophylla* Polymer/Nanocomposites for Mitigating Paraffin Wax Deposition

**DOI:** 10.3390/polym18151898

**Published:** 2026-08-02

**Authors:** Abubakar Aji, Mysara Eissa Mohyaldinn, Hisham Khaled Ben Mahmud, Abdullah Abduljabbar, Ibnelwaleed A. Hussein

**Affiliations:** 1 Department of Petroleum Engineering, Universiti Teknologi PETRONAS (UTP), Seri Iskandar 32610, Perak Darul Ridzuan, Malaysia; abduljabbar.ahmed@utp.edu.my; 2 Center of Flow Assurance, Institute of Sustainable Energy & Resources, Universiti Teknologi PETRONAS (UTP), Seri Iskandar 32610, Perak Darul Ridzuan, Malaysia; 3 Faculty of Engineering, Sohar University, Sohar 311, Oman; hmahmud@su.edu.om; 4 Gas Processing Center, College of Engineering, Qatar University, Doha P.O. Box 2713, Qatar; ihussein@qu.edu.qa; 5 Chemical Engineering Department, College of Engineering, Qatar University, Doha P.O. Box 2713, Qatar

**Keywords:** metal nanocomposites, paraffin wax, pour point depressant (PPD), *Swietenia macrophylla*

## Abstract

Paraffin wax precipitation and deposition significantly hinder crude oil production and transportation by reducing flow efficiency, increasing operational downtime, and requiring costly remediation procedures. Conventional wax mitigation methods commonly rely on environmentally unfriendly chemicals and energy-intensive thermal or mechanical treatments. This study investigates the use of a natural polymer from *Swietenia macrophylla* (Mahogany), modified with metal nanoparticles (MNPs), namely silver oxide (Ag_2_O), zinc oxide (ZnO), and silver-doped zinc oxide (Ag-ZnO), for petroleum wax inhibition. Material characterization was conducted using FTIR, TGA and GC-MS analyses, while performance evaluation employed rheological measurements, Cross-Polarized Microscopy (CPM), and pour point testing. GC-MS analysis revealed the presence of oxygenated fatty acid esters such as glycidyl oleate (59.61%) and glycidyl palmitate (16.86%). These compounds indicate the presence of hydrocarbon-compatible and surface-active constituents beneficial for wax crystal modification. FTIR spectra further confirmed carbonyl, aliphatic hydrocarbon, and ether functionalities associated with natural wax-mitigation compounds and effective MNP binding sites. The application of 2 wt% polymeric materials demonstrated significant wax inhibition performance. The unmodified polymer reduced the activation energy (Ea) for crude oil flow by 49.4 kJ·mol^−1^ from the virgin crude oil value of 213.6 kJ·mol^−1^. The polymer + ZnO formulation achieved the highest pour point reduction of 1.72 °C, while polymer + Ag-ZnO recorded the greatest viscosity reduction of 77.1% at 1 s^−1^ and 91.6% at 200 s^−1^. This study demonstrates, for the first time, the potential of *Swietenia macrophylla*-derived nanocomposite polymers as sustainable and effective wax mitigation agents for waxy crude oil systems.

## 1. Introduction

The persistent challenge of paraffin wax deposition within crude oil production and transportation infrastructure represents a critical operational bottleneck in the petroleum industry worldwide. As crude oil is extracted and transported, the natural decrease in temperature and pressure causes long-chain hydrocarbon components (paraffin waxes) to precipitate from the liquid phase. This precipitation leads to the formation of a rigid gel structure, commonly known as wax buildup, which significantly increases the crude oil’s viscosity, elevates the pour point, and severely restricts flow assurance [1]. The resulting reduction in pipeline throughput, coupled with the need for frequent, costly mechanical and chemical remediation (such as pigging, hot oil circulation, and solvent treatments), leads to substantial economic losses and increased environmental risk [2].

Common treatment methods for wax deposition include thermal methods, chemical additives, mechanical pigging, and advanced materials and technologies. Each of these methods has advantages and disadvantages. If wax deposition is not handled properly, it can pose risks to the environment and operational safety [3]. For example, the thermal method is energy-intensive, and chemicals such as ethylene-vinyl acetate (EVA) are synthesized from non-renewable petrochemical sources and often exhibit insufficient thermal stability. Research has confirmed that the use of toxic and non-ecofriendly chemicals is a major contributor to environmental pollution and the spread of diseases [4].

In response to this growing demand, recent research has focused on the use of sustainable, bio-derived materials and their integration with advanced nanotechnology. Bio-derived polymers, owing to their inherent biodegradability, low toxicity, and renewable feedstocks, offer a compelling path toward green chemical solutions. Specifically, natural plant polymers, rich in complex biopolymer structures, have shown promise as foundational materials for synthesizing novel flow improvers [5].

This study focuses on a sustainable polymer derived from the seed pods of *S. macrophylla* (Mahogany), an abundant tropical resource. The intrinsic chemical structure of this bio-derived polymer, which may contain long-chain fatty esters, polyesters, or other natural macromolecular architectures, is hypothesized to directly interfere with the nucleation and growth kinetics of paraffin wax crystals. As a non-edible polymer, it avoids competition with food resources, making it a sustainable option. However, the performance of neat biopolymers in industrial applications is often limited by their mechanical strength and stability under harsh field conditions [6].

To surmount these limitations and enhance the functional properties of the bio-derived material, this research integrates the *S. macrophylla* polymer with metal nanoparticles (MNPs) to form nanocomposites. While previously reported edible biopolymers raise food security concerns [7], and polymer–nanoparticle hybrid flow improvers (such as EVA–silica) are almost exclusively synthesized from non-renewable, petrochemical matrices that pose toxicological and environmental risks, the literature reports on bio-derived flow assurance modifiers remain extremely limited. To date, the literature reports on bio-derived flow assurance modifiers remain extremely sparse, and there is no documented application of a *S. macrophylla* seed pod polymer, either alone or hybridized with metallic/metal oxide nanoparticles (especially bimetallic Ag-ZnO), for paraffin wax mitigation. By integrating this sustainable bio-derived polymer, which is rich in natural fatty acid esters (e.g., glycidyl oleate) that possess excellent wax compatibility and surfactant-like properties, with green-synthesized metal and metal oxide nanoparticles (Ag_2_O, ZnO, and Ag-ZnO), this study presents a green, non-toxic nanocomposite flow modifier that circumvents the ecological drawbacks of synthetic polymer matrices while providing robust viscosity reduction [8].

The primary objective of this investigation is therefore to synthesize, fully characterize, and systematically evaluate the efficacy of the *S. macrophylla*-derived polymer/MNPs nanocomposites as a high-performance PPD and wax growth inhibitor. This paper details the synthesis procedure, structural characterization using FTIR, XRD, GC-MS, and a comprehensive performance assessment utilizing standard rheological measurements, Cross-Polarized Microscopy (CPM), and pour point determination. This work aims to establish the potential of these novel, sustainable nanocomposites as a superior, eco-friendly solution for mitigating paraffinic challenges in the upstream and midstream petroleum sectors.

## 2. Materials and Methods

### 2.1. Material

The crude oil samples used in this investigation were Dulang crude oil and were characterized for their wax content and physical properties. 

The *S. macrophylla* (Mahogany) seed polymer was purchased from the commercial market in Maiduguri, Borno State, Nigeria. This polymer was pre-refined using a vacuum filtration setup with Whatman filter paper to remove the suspended material. This was then kept in the laboratory before analysis. Ag_2_O, Ag-ZnO, and ZnO were obtained from the green-synthesized nanomaterial as mentioned in [9].

### 2.2. Development of S. macrophylla Nanomaterial

Hybridization of the Ag_2_O, Ag-ZnO, and ZnO components was achieved through ultrasonic homogenization, adapting the methodology reported by [10]. The instrument parameters were set to 500 W of power, a 10 min operation time, a 5.0 s ‘on’ interval, a 0.05 s ‘off’ interval, and a temperature of 70 °C. A nanomaterial concentration of 0.04 wt% was adopted because previously it was identified as effective by Hamidian et al. [11]. Ultrasonication is a widely accepted and standardized technique for the effective redispersion of nanopowders in liquid media and the homogenization of nanoparticle suspensions. A primary objective of this sonication process is to disaggregate agglomerates without compromising the inherent physicochemical properties of the primary particles [12]. Subsequently, the mixture was cured in an oven at 40 °C for 40 min.

The presence of endogenous capping agents, including oleic acid, linoleic acid, palmitic acid, stearic acid, and trace amounts of other fatty acids, as detailed in [13], is anticipated to facilitate the uniform dispersion and blending of the Np with the polymer matrix.

### 2.3. Characterization Techniques

A wax content analyzer was used for the analysis of the wax content in the crude oil. For the Dulong crude oil and *S. macrophylla* polymer, FTIR and GC-MS were used to elucidate the compounds.

### 2.4. Crude Oil Application of Polymer Nanomaterials

To ensure an accurate investigation of the interaction between crude oil and inhibitors, proper sample preparation was required. Initially, one hundred grams (100 g) of Dulang crude oil were measured into a beaker. This sample was then preheated in an oven at 70 °C for 90 min (1.5 h). This preheating step was crucial to ensure that any wax that had previously crystallized in the oil was completely dissolved [14]. Subsequently, 2 wt% of the synthesized nanoparticles were blended with the crude oil using the ultrasonicator settings mentioned above [15]. A polymer NM concentration of 2wt% was adopted as previously utilized by Yan et al. [16].

In the present study, the addition levels were fixed at 2 wt% polymer and 0.04 wt% nanoparticles to provide a controlled formulation for evaluating the interaction of the synthesized polymer nanocomposite with Dulang crude oil under identical preparation and testing conditions. This fixed ratio was selected because recent studies show that nanocomposite performance is highly system-dependent and varies with polymer composition, nanoparticle content, crude oil composition, wax content, and treatment conditions [16]. The nanoparticle loading of 0.04 wt% was adopted based on the previously optimized nanomaterial preparation condition described above, whereas the 2 wt% polymer loading was chosen, despite being used in the literature, to ensure sufficient active material for homogeneous dispersion and measurable flow improvement response in the crude oil system. Accordingly, the objective of the present work was to establish the efficacy of the selected polymer nanoparticle formulation, rather than to determine the universal optimum dosage [15,16]. This dosage optimization aspect was recommended for future work through systematic variation of both polymer and nanoparticle concentrations. In the present work, pure Dulang crude oil was deliberately selected as the model crude oil system. Dulang crude is recognized in Malaysia as one of the most wax-rich crudes, with a wax appearance temperature (WAT) and pour point above ambient conditions. At room temperature, it exhibits semi-solid, crystalline behavior due to its high paraffinic content and elevated wax fraction. This unique property makes Dulang crude particularly suitable for flow assurance studies, as it represents the most severe wax-related challenges encountered in Malaysian production fields [17,18].

### 2.5. Rheological Measurements and WAT

Rheological measurements, specifically temperature sweep and flow sweep tests, were conducted using a rheometer to investigate how polymer nanomaterial inhibitors affect the flow properties of Dulang crude oil under various conditions. Rheometers complement PLM by measuring rheological properties such as viscosity, yield stress, and viscoelastic moduli under controlled shear rates and temperatures [19].

To determine the WAT, temperature sweep tests were performed on the crude oil. These tests involved heating and cooling the oil between 14 °C and 59 °C, under varying shear rates (1/s to 200/s), to pinpoint the temperature at which wax began to precipitate. Below the WAT, a significant increase in viscosity occurred as the crude oil’s behavior shifted from Newtonian to non-Newtonian. This critical point, where the transition between Newtonian and non-Newtonian shear-thinning behaviors takes place, is defined as the abnormal point [18]. The inhibitors’ efficacy is confirmed by their successful reduction of crude oil viscosity, especially in the low-temperature range where non-Newtonian behavior is characteristic [20].

The Temperature-Dependent Viscosity (TD − VR) index is calculated using Equation (1) below.

(1) $$TD-VR\;\left(T\right)=\frac{\mu 0\left(T\right)-\mu t\left(T\right)}{\mu 0\left(T\right)}$$

While the Viscosity Reduction is calculated by Equation (2) below

(2) $$PIE\;\left(\%\right)=\frac{\mu Virgin\left(T\right)-\mu treated\left(T\right)}{\mu Virgin\left(T\right)}\;\times \;100$$

where:

μ Virgin Viscosity of untreated crude at temperature T

μ Treated Viscosity of treated crude at temperature T

### 2.6. Pour Point Determination

The pour point (PP) of untreated and treated crude oil samples was determined using the ASTM D97 Bottle Test Method, the standard procedure for evaluating the lowest temperature at which petroleum products remain capable of flowing. This method employs a controlled cooling protocol to establish the pour point with precision and reproducibility.

For each test, approximately 45 mL of crude oil, virgin or containing the inhibitor formulation, was introduced into a standard glass jar with a thermometer and stopper. The samples were initially heated to 70 °C to ensure complete dissolution of wax crystals, thereby standardizing the starting condition before cooling. The jars were subsequently placed in a constant-temperature bath. At each temperature interval, the jars were removed and tilted horizontally to assess flow behavior. The pour point was defined as the temperature at which the sample ceased to flow, and the final PP value was calculated as the temperature at which flow was last observed [21,22]. This standardized approach ensures consistency in evaluating the effectiveness of polymeric and nanocomposite additives in pour point depression studies.

### 2.7. Cross-Polarized Microscopy (CPM)

Cross-Polarized Microscopy (CPM) was employed as a qualitative and quantitative technique to directly observe the morphology, size, and aggregation behavior of paraffin wax crystals in the Dulang crude oil samples, both untreated and treated with the *S. macrophylla* polymer/MNPs nanocomposites. This technique utilizes the birefringent properties of wax crystals, which appear bright against a dark background under crossed polarizers, to visualize the phase transition. For this study, a temperature-controlled stage was used to cool the crude oil samples from 60 °C (above the WAT) to 25 °C at a controlled rate. Micrographs were captured at specific temperatures (notably near the WAT and at 25 °C) to assess the onset of wax crystallization and the inhibitory effect of the additives on crystal growth and network formation. The results from CPM provide direct visual confirmation of the pour point depression and rheological modification mechanisms [23].

### 2.8. Digital Image Processing of WAT Micrographs

Cross-Polarized Microscopy (CPM) images of Dulang crude oil and inhibitor-treated samples at the WAT were analyzed using an automated Python-based pipeline within the Antigravity (v1.22.2) environment. The workflow involved grayscale conversion of RGB micrographs (1532 × 2048 pixels) into 8-bit space, adaptive Otsu thresholding to segment birefringent wax crystals from the oil matrix, and morphological cleaning with a 3 × 3 structural matrix to remove high-frequency noise [24]. Morphometric quantification was performed using SciPy’s ndimage filter to isolate, count, and calculate the average surface area of individual crystals, enabling comparative evaluation of polymeric and polymer nanoparticle formulations against untreated crude oil [25].

## 3. Results and Discussion

The results obtained from this study unveiled the influence of *S. macrophylla* polymer and its metal nanocomposites on the flow assurance properties of Dulang crude oil. Table 1 presents the principal physical and chemical properties of the Dulang crude oil sample used in this investigation, providing essential baseline data for assessing the performance of the synthesized inhibitors. The results underscore the waxy nature of the crude, as evidenced by a relatively elevated wax appearance temperature (WAT) of 33.4 °C and a pour point (PP) of 33.25 °C, measured in this study. The high PP corroborates the crude’s pronounced tendency to wax gel at near-ambient conditions, thereby posing a significant flow-assurance challenge.

Further compositional analysis indicates a wax fraction of 19.5 wt%. The markedly high viscosity at 25 °C (24,510.7 cP) is attributable to the elevated paraffin concentration and the resultant wax network formation. The crude’s physical properties, including an API gravity of 37.39 and a density of 0.837 g/cm^3^, classify Dulang crude oil as a light, waxy crude. Collectively, these intrinsic characteristics underscore the need for effective pour point depression and wax mitigation strategies, against which the efficacy of the *S. macrophylla*-derived nanocomposites is systematically benchmarked.

### 3.1. Analytical Characterization of S. macrophylla by FTIR and GC–MS

**Figure 1:**
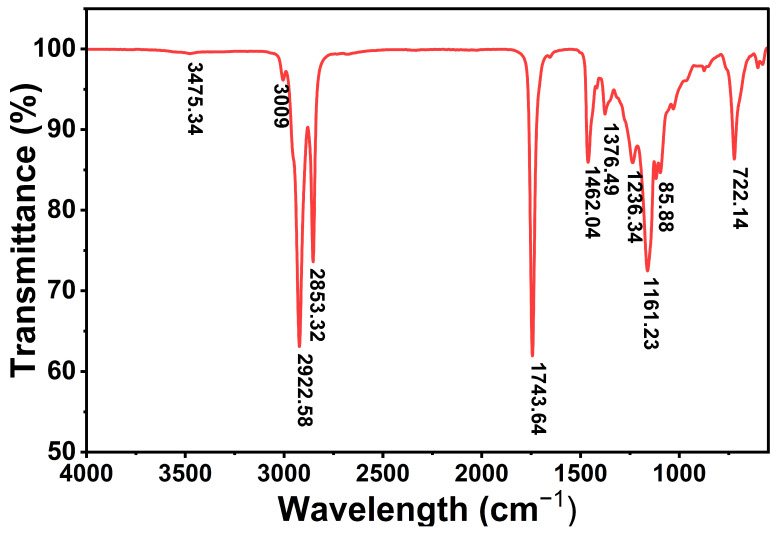
FTIR spectrum of *S. macrophylla*.

The FTIR spectral profile of *S. macrophylla* reveals the presence of various functional groups that are commonly associated with phytochemicals, including flavonoids, terpenoids, saponins, and phenolics (Figure 1).

**Figure 2:**
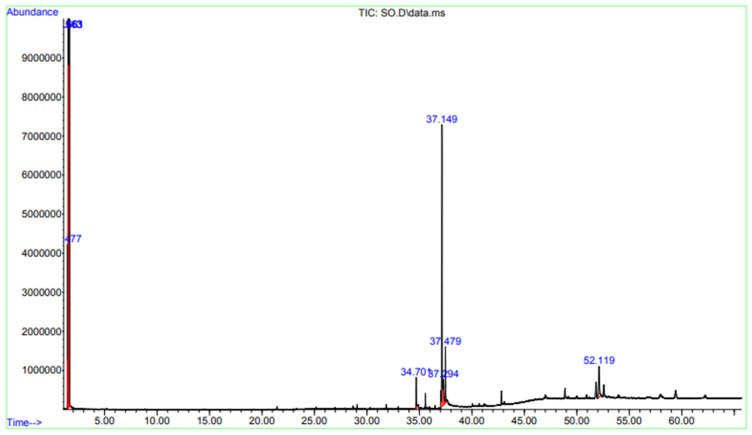
GC–MS total ion chromatogram (TIC) of *S. macrophylla*, showing the relative abundance of detected compounds.

Notably, the strong C=O stretch at 1743.64 cm^−1^ indicates the presence of carbonyl-containing compounds such as aldehydes, ketones, esters, or carboxylic acids [27]. C–H stretching bands around 2922.58 cm^−1^ and 2853.62 cm^−1^ confirm aliphatic hydrocarbon chains, possibly derived from lipids or fatty acid components [28]. Oxygen-containing groups (C–O stretches) around 1236.34 cm^−1^, 1161.23 cm^−1^, and 1117.81 cm^−1^ suggest the presence of alcohols, ethers, or ester functionalities, likely from plant secondary metabolites such as polysaccharides and phenolic compounds [29]. The GC–MS analysis of *S. macrophylla* polymer revealed a complex chemical profile comprising both volatile hydrocarbons and higher molecular weight oxygenated compounds (Figure 2). Nine major peaks were detected across the chromatogram, with retention times ranging from 1.477 to 52.119 min. The corrected peak areas indicate that the most abundant constituents eluted at 1.513 min (34.92% relative area), 1.563 min (13.88%), and 37.149 min (21.16%).

Early-eluting peaks 1 to 4 (RT 1.477–1.661–37.479 min) constitute carrier solvents (Pentane and n-Hexane isomers). After isolating the active organic fraction and normalizing it to a solvent-free distribution, the peaks (RT 34.701–37.479 min) were characterized by long-chain fatty acid derivatives, notably glycidyl palmitate and glycidyl oleate, and by unsaturated aldehydes such as (Z)-9-octadecenal. The detection of these compounds is consistent with lipid-derived constituents commonly reported in mahogany species and associated with bioactive properties, including antioxidant and antimicrobial activities. The relatively high abundance of glycidyl oleate (RT 37.149 min) underscores the prominence of unsaturated fatty acid esters within the polymer (Table 2).

The late-eluting peak at 52.119 min comprised complex oxygenated molecules, including dioxolane derivatives and substituted cyclohexenes. These compounds indicate the presence of resinous or polymeric fractions that may contribute to the polymer’s stability and potential industrial applications.

Hence, the GC–MS profile demonstrates that the *S. macrophylla* seed polymer is chemically diverse. This reveals that Glycidyl oleate (59.61%) and Glycidyl palmitate (16.86% combined) are the primary active capping and modifying esters of the seed oil matrix. These findings align with previous reports on mahogany phytochemistry [13] and support the potential utility of the *S. macrophylla* polymer in applications such as wax inhibition, antioxidant formulations, and surfactant development.

The pour point (PP) depression behavior of Dulang waxy crude oil (VCO) in the presence of a bio-derived polymer and its nanocomposites exhibits distinct performance trends. The baseline crude oil exhibited a PP of 33.25 °C, which served as the reference for all subsequent additive formulations.

### 3.2. Thermal Stability of Polymer Nanocomposites for Wax Inhibition

**Figure 3:**
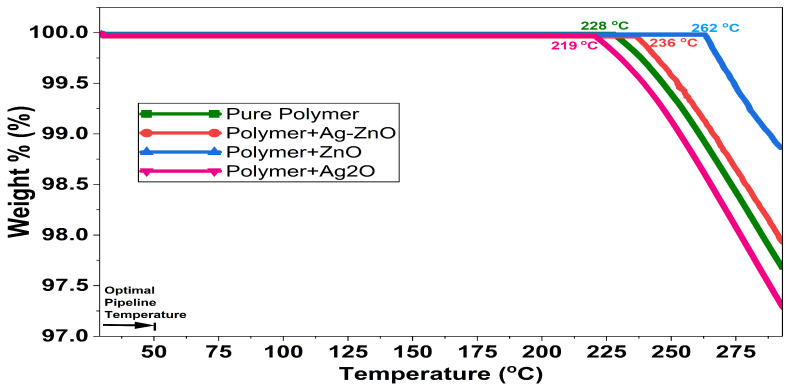
Thermogravimetric analysis (TGA) of polymer nanocomposites for wax inhibition.

In the thermogravimetric analysis (TGA) of the polymer nanocomposites designed for wax inhibition (Figure 3), all samples demonstrated remarkable thermal endurance well above the average pipeline operating temperature of 50 °C [30,31]. The pure polymer exhibited an initial degradation onset at approximately 228 °C, retaining more than 97–99% of its weight up to 200 °C. Incorporation of Ag-ZnO and ZnO nanoparticles further enhanced the thermal stability, shifting the onset to ~236 °C and ~262 °C, respectively. This improvement reflects the reinforcing effect of these nanomaterials, which delay polymer chain scission and provide stronger interfacial bonding. In contrast, the Ag_2_O composite showed an earlier onset at ~219 °C, indicating reduced stability compared to the unblended polymer, likely due to weaker polymer–nanoparticle interactions. The nanomaterials (except Ag_2_O) significantly increased the thermal stability of the polymer matrix, ensuring that the composites withstand pipeline conditions with >97% weight retention. Among them, ZnO proved to be the most effective stabilizer, while Ag-ZnO offered balanced performance between wax inhibition and thermal resistance. This confirms that nanoparticle incorporation, particularly ZnO, is a promising strategy for enhancing the durability of polymer-based wax inhibitors under operational stresses [32,33].

Incorporation of the bio-derived polymer alone at 2% concentration produced a modest improvement, lowering the PP to 31.7 °C. This 1.55 °C (4.66%) reduction indicates that the polymer functions as a wax crystal modifier, partially inhibiting the growth of large paraffin crystals and thereby enhancing crude oil flowability. The most pronounced effect was achieved with the combined formulation of 2% polymer and 0.04% ZnO nanoparticles (Table 3. This system reduced the PP to 31.53 °C, corresponding to a 1.72 °C decrease (5.17%). The superior performance suggests a synergistic interaction between the polymer and ZnO nanoparticles, likely through enhanced nucleation or improved dispersion that facilitates more effective modification of wax crystal structures.

By contrast, silver-based nanocomposites demonstrated limited efficacy. The Ag_2_O formulation yielded a PP of 32.75 °C, representing only a 0.5 °C reduction from the baseline. Similarly, the bimetallic Ag-ZnO composite produced a PP of 31.9 °C, a 1.35 °C reduction that was inferior to both the polymer-only and ZnO-based systems. These results imply that silver incorporation may interfere with the polymer–wax interaction, potentially promoting unfavorable crystal growth.

Hence, the findings establish the polymer–ZnO nanoparticle formulation as the optimal pour point depressant for Dulang crude oil. The achieved PP of 31.53 °C underscores the effectiveness of ZnO nanoparticles as synergists, offering a promising strategy for mitigating flow assurance challenges in waxy crude systems [8].

### 3.3. Rheological Analysis

- **Flow sweep**

**Figure 4:**
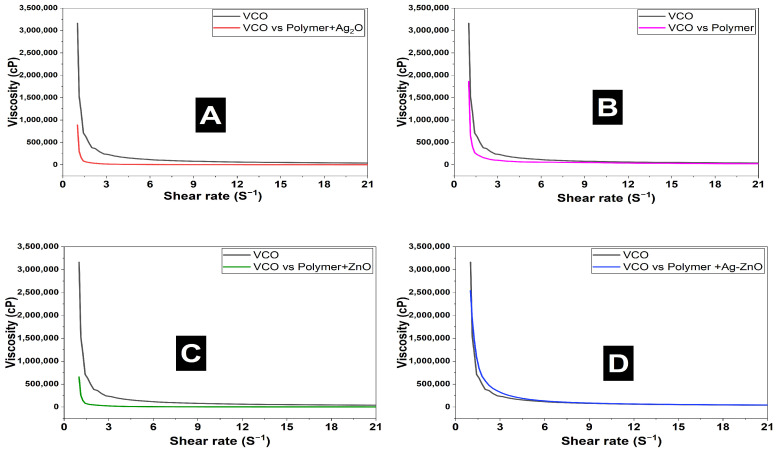
Viscosity–shear rate behavior of virgin crude oil (VCO) and crude oil treated with S. macrophylla polymer and metal nanocomposites (2% polymer + 0.04% MNP) at constant temperature: (**A**) VCO versus Polymer + Ag_2_O; (**B**) VCO versus Polymer; (**C**) VCO versus Polymer + ZnO; (**D**) VCO versus Polymer + Ag-ZnO.

Flow sweep tests were conducted to further examine how well polymer-based nanomaterials reduce crude oil viscosity. Viscosity versus shear rate (ranging from 1 s^−1^ to 200 s^−1^) was measured at two temperatures: 30 °C (near the pour point temperature, PPT) and 20 °C (below the PPT). Analysis of untreated and treated crude oils at 20 °C and 30 °C consistently demonstrated that viscosity continuously decreased as the shear rate increased (Figure 4).

(I) **Flow sweep at 20 °C**

At 20 °C, the virgin crude oil (Dulang) without modifiers exhibited extremely high viscosity at low shear rates, with values of 3.16 × 10^6^ cP at 1 s^−1^, decreasing to 1.10 × 10^4^ cP at 200 s^−1^ (Figure 4). This pronounced shear-thinning behaviour reflects the strong structural resistance of wax crystal networks, which impede flow initiation and pipeline startup.

The introduction of 2% *S. macrophylla* polymer significantly reduced viscosity. At 1 s^−1^, viscosity dropped to 1.86 × 10^6^ cP, reducing the viscosity by 1.3 × 10^6^ cP. At 200 s^−1^, viscosity further decreased to 2339 cP (Table 4), confirming the polymer’s ability to disrupt wax crystal aggregation and improve flowability (Figure 4B).

The polymer + ZnO material also demonstrated enhanced viscosity suppression. At 1 s^−1^, viscosity was 6.6 × 10^5^ cP, and at 200 s^−1^, it reached just 305.79 cP. Compared to the polymer-only system, ZnO incorporation yielded 63% lower viscosity at low 1 (S^−1^) shear, 92.2% at 10 (S^−1^) shear, and 86.9% inhibition at 200 (S^−1^) shear, indicating improved inhibition of wax networking (Figure 4C).

Ag_2_O addition also produced a pronounced effect. At 1 s^−1^, viscosity was reduced to 2.54 × 10^6^ cP, while at 200 s^−1^, it reached 3.26 × 10^2^ cP. This represents a ~19.6% and ≈97.1% reduction relative to the VCO at 1 and 200 s^−1^ shear rate, respectively, highlighting Ag_2_O’s superior dispersive capacity in destabilizing wax crystal growth (Figure 4A).

The Ag-ZnO hybrid nanocomposite exhibited the most significant improvement. At 1 s^−1^, viscosity was 7.24 × 10^5^ cP, and at 200 s^−1^, it dropped to 9.12 × 10^2^ cP. This is equivalent to a 77.1% and 91.6% reduction in viscosity in VCO at 1 s^−1^ and 200 s^−1^, respectively (Table 4). The synergistic effect of Ag doping enhances particle–polymer interactions, yielding superior wax inhibition and rheological stabilization [34] (Figure 4D).

The comparative rheology confirms that the bio-derived polymer alone substantially improves crude oil flowability, but the incorporation of metal oxide nanocomposites amplifies performance (Table 4). These results validate the hypothesis that sustainable, bio-derived polymers reinforced with nanotechnology can serve as highly effective pour point depressants (PPDs) and wax inhibitors, offering environmental compatibility and technical efficacy [35].

(II) **Flow sweep at 30 °C**

**Figure 5:**
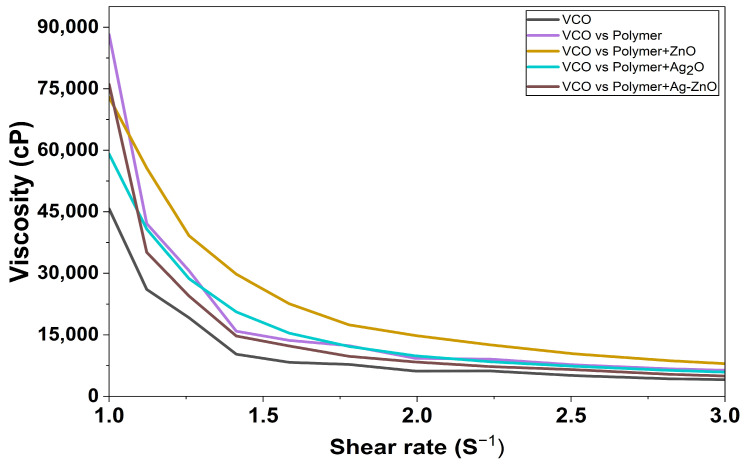
Flow sweep of VCO and wax inhibitors at 30 °C.

The flow sweep behavior of Dulang crude oil and its modified systems at 30 °C reveals important rheological insights that are central to understanding wax deposition and inhibitor performance (Figure 5).

The introduction of polymeric and nanoparticle-based modifiers at 30 °C does not reduce viscosity at this temperature; rather, they increase it. The polymer alone enhances viscosity, suggesting that it interacts with wax crystals to reinforce the gel-like network. When combined with Ag_2_O, the structuring effect is even more pronounced, indicating that Ag_2_O nanoparticles promote further aggregation or stabilization of wax–polymer complexes near a WAT of 30 °C. The hybrid Ag-ZnO system produces the highest viscosity. Such behavior implies that these modifiers act as structuring agents at 30 °C rather than dispersants, which may be beneficial in certain storage or stability contexts but detrimental for pipeline transport.

From a flow assurance perspective, the findings emphasize that inhibitor performance is highly temperature-dependent and shear-dependent. At 30 °C, the modifiers reinforce viscosity rather than reduce it, as compared to at 20 °C. Their effectiveness may only be apparent at higher temperatures or under dynamic flow regimes where wax crystal growth and network formation are more readily disrupted. This underscores the need for careful optimization of inhibitor design, balancing structural reinforcement with flowability [5].

ii. **Temperature Sweep**

**Figure 6:**
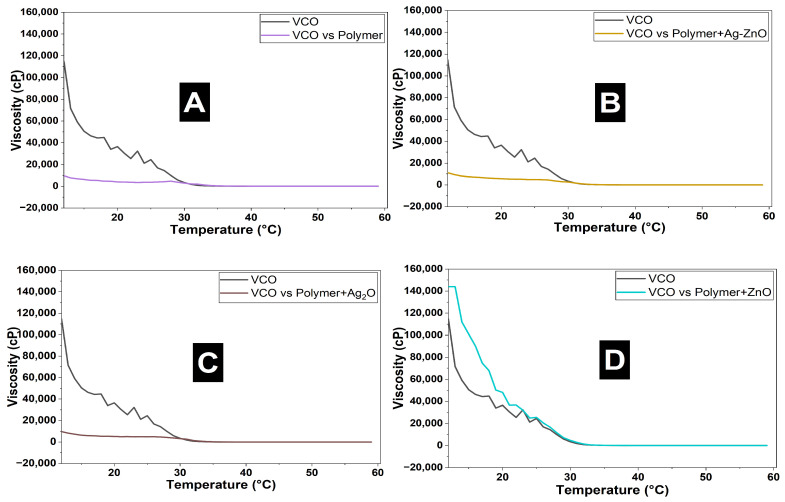
Temperature sweep rheology of the different wax modifiers plots of (viscosity versus temperature): (**A**) VCO vs. polymer, (**B**) VCO vs. polymer + Ag-ZnO, (**C**) VCO vs. polymer + Ag_2_O and (**D**) VCO vs. polymer + ZnO.

A temperature sweep of the rheology analyses of the VCO and the different additives’ flow behavior is captured in Figure 6.

The viscosity of VCO exhibits a strong temperature dependence, decreasing from 114,837 cP at 12 °C to 3.57 cP at 53 °C. This sharp decline reflects the typical shear-thinning and thermal-softening behavior of waxy oils, where crystalline structures dominate at low temperatures but progressively melt and disaggregate with increasing thermal energy.

Upon incorporation of the polymer, the viscosity profile is markedly altered, as can be seen in Figure 6A. At 12 °C, the VCO + polymer records 9608.69 cP, representing a reduction of nearly 92% relative to VCO. This substantial decrease indicates that the polymer disrupts wax crystal networking, thereby enhancing flowability under cold-start conditions. This trend persists across the temperature sweep. At 20 °C, viscosity falls from 36,447.6 cP (VCO) to 3956.37 cP (VCO + polymer), and at 30 °C, from 3401.45 cP to 2859.09 cP. The polymer acts as a rheology modifier, reducing viscosity across all temperatures tested. Its effect is most pronounced in the low-to-mid temperature regime (12–25 °C), where wax deposition and crystal growth are typically most problematic.

The addition of polymer + Ag-ZnO markedly reduces viscosity to 11,149.3 cP, representing a >90% reduction. This demonstrates the composite’s efficiency in disrupting wax networks and enhancing flowability; Figure 6B.

Across the mid-temperature range, the modified system consistently maintains lower viscosity values. For example, at 20 °C, VCO records 36,447.6 cP, while the modified sample shows 5684.93 cP. At 30 °C, viscosity decreases from 3401.45 cP (VCO) to 2638.08 cP (VCO + polymer + Ag-ZnO). These reductions confirm the stabilizing effect of the additive, which suppresses wax structuring and reduces shear resistance [36].

**Figure 7:**
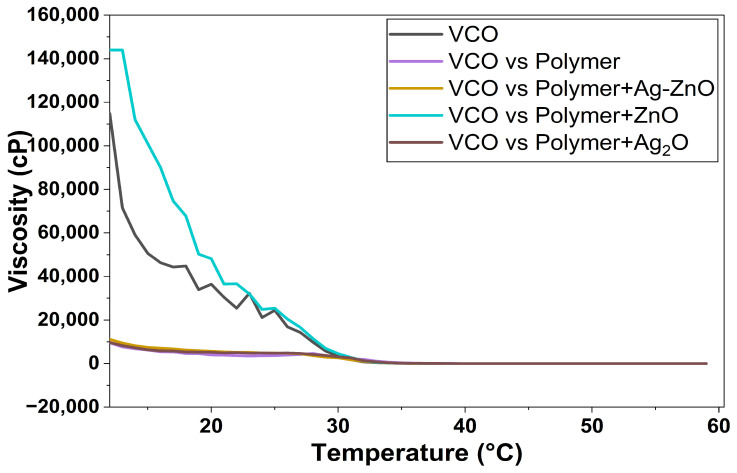
Combined comparison of the different wax modifier plots (viscosity versus temperature).

At elevated temperatures (>40 °C), both systems converge toward minimal viscosity values. However, the modified oil continues to exhibit smoother flow, e.g., 19.35 cP vs. 17.05 cP at 40 °C, and 3.80 cP vs. 4.72 cP at 59 °C, indicating residual stabilization and prevention of microstructural reformation (Figure 7).

Upon incorporation of polymer + Ag_2_O, the viscosity profile is significantly altered. At 12 °C, the modified system records 9715.02 cP, indicating a reduction of > 91%, which confirms the additive’s efficiency in suppressing wax structuring and enhancing flowability under cold-start conditions, seen in Figure 6C. Across the mid-temperature range, the modified system maintains consistently lower viscosity values compared to neat VCO. For instance, at 20 °C, VCO records 34,647.6 cP, while the modified sample shows 5175.91 cP. At 30 °C, the reduction continues from 3401.45 cP (VCO) to 3104.8 cP (VCO + polymer + Ag_2_O). These results suggest that the additive not only disrupts wax crystal formation but also stabilizes the fluid matrix, reducing interfacial resistance and improving shear response [8]. At elevated temperatures (>40 °C), both systems converge toward minimal viscosity values, yet the modified oil continues to exhibit smoother flow. For example, at 59 °C, VCO records 4.72 cP, while the modified system shows 5.75 cP.

When polymer + ZnO is introduced, the viscosity profile changes significantly. At low temperatures, the modified system records markedly higher viscosities, such as 143,963 cP at 12–13 °C and 67,803.1 cP at 18 °C, indicating that ZnO in combination with the polymer promotes structural reinforcement or agglomeration rather than inhibition (see Figure 6D).

As the temperature increases, the modified system shows progressive viscosity reduction, although values remain consistently higher than neat VCO across most of the sweep. For example, at 30 °C, VCO records 3401.45 cP, while the modified system shows 4558.06 cP. At 40 °C, the difference narrows to 17.05 cP (VCO) versus 20.47 cP (VCO + polymer + ZnO), and by 59 °C, both systems converge, with 4.72 cP for VCO and 5.28 cP for the modified system. This behavior suggests that polymer + ZnO does not act as a viscosity-reducing agent in the same manner as polymer alone or polymer + Ag-ZnO. Instead, it appears to induce a transient increase in rigidity at low temperatures, possibly due to ZnO’s surface activity and its interaction with polar groups in the polymer matrix.

Hence, all modifiers act by interfering with wax crystallization and aggregation, but their efficiency varies. Polymer alone and polymer + Ag_2_O provide strong and consistent viscosity reduction, making them effective cold-start flow improvers. Polymer + Ag-ZnO demonstrates synergistic inhibition, offering broad-range stability and enhanced suppression. Polymer + ZnO, however, shows limited utility at low temperatures due to viscosity elevation, though it contributes to stabilization at higher temperatures. These findings highlight the importance of additive selection and nanoparticle chemistry in tailoring rheological modification strategies for waxy crude oils [36] (Figure 7).

### 3.4. Percentage Inhibition Efficiencies of Wax Modifiers in Dulang Waxy Crude Oil

The comparative viscosity profiles of Dulang crude oil treated with *Swietenia macrophylla* polymer nanocomposites reveal distinct performance trends across the temperature range of 12–31 °C (Table 5). The polymer + Ag-ZnO system consistently maintained high inhibition efficiencies (>85%) between 12 and 20 °C, demonstrating its superior ability to suppress wax crystal networking under near-ambient conditions. This behavior is indicative of strong polymer–nanoparticle synergy, where Ag doping enhances particle dispersion and interfacial interactions, thereby yielding smaller, less interconnected wax crystals and improved flowability.

In contrast, the polymer + ZnO system exhibited anomalous negative values at several temperatures, particularly between 13 and 19 °C. These apparent viscosity increases are not mechanistic but rather artifacts of rheometer sensitivity and baseline drift at low viscosities. Such deviations are well-documented in waxy crude rheology, where instrument resolution can exaggerate small differences. Nonetheless, the ZnO system still demonstrated effective inhibition at higher temperatures (>20 °C), confirming its role as a synergist in pour point depression.

The polymer + Ag_2_O formulation showed moderate inhibition, with efficiencies ranging from 70 to 90% across most of the tested temperatures. While silver oxide incorporation improved dispersion capacity, its performance was less consistent than Ag-ZnO, suggesting that bimetallic synergy is more effective than monometallic silver systems in destabilizing wax crystal growth.

At elevated temperatures (>25 °C), all systems exhibited declining inhibition values, with some negative readings. This decline reflects the reduced relevance of wax inhibition once the crude transitions into a Newtonian regime, where wax crystals are largely dissolved. The negative values should therefore be interpreted as experimental noise rather than true viscosity increases.

Analysis of the comparative viscosity data demonstrates that the most effective inhibition occurs within the temperature window of 12–20 °C, where the Ag-ZnO nanocomposite consistently achieved efficiencies above 85–90%. This interval corresponds to the critical wax precipitation zone of Dulang crude oil, in which paraffin crystallization is most pronounced and flow assurance challenges are most severe. Effective mitigation strategies should therefore prioritize maintaining crude oil within this operationally minimal temperature range, combining inhibitor deployment with targeted thermal management to suppress wax nucleation while avoiding destabilization regimes at higher temperatures [37].

**Table 5:** Comparative performance of *S. macrophylla* polymer nanocomposites and reported silica-based systems for wax inhibition.

Entries	Material/System	Performance Metrics	Reference
1	Polymer	% Inhibition = 91.63 at 12 °C	Present work
2	Polymer + Ag_2_O	% Inhibition = 91.54 at 12 °C	Present work
3	Polymer + Ag-ZnO	% Inhibition = 90.29 at 12 °C	Present work
4	Polymer + ZnO	% Inhibition = −25.36 at 12 °C	Present work
5	Silica nanoparticles (pomegranate peel)	60% decrease in wax thickness (pipeline simulation)	[38]
6	Modified nanosilica in terpolymer nanohybrids	Up to 71% paraffin inhibition, 83% viscosity reduction, 9 °C pour point reduction	[39]
7	EVA/SiO_2_ nanocomposite	PP and viscosity reduced by 17 °C and 83.9% at 25 °C, respectively.	[40]
8	Modified silica flow improver	At 100 ppm, viscosity reduction ≥ 60%	[41]

### 3.5. Activation Energy Trend

**Figure 8:**
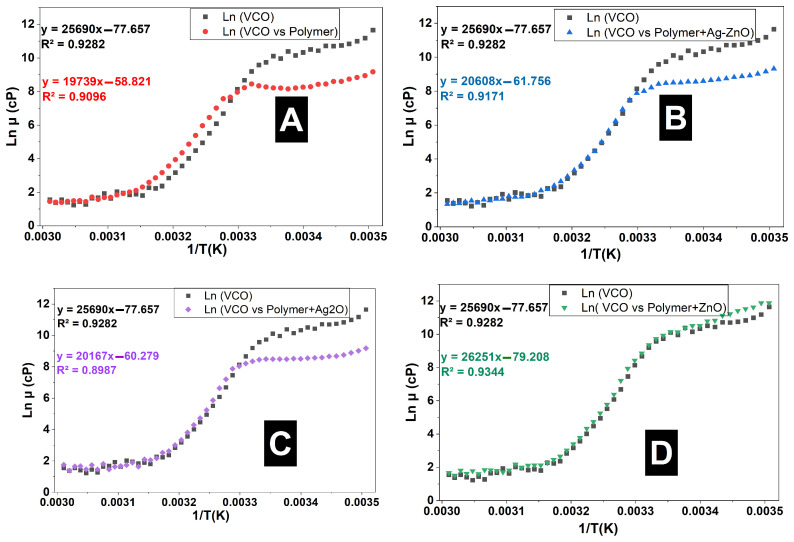
Arrhenius plots of Ln(VCO) versus reciprocal temperature (1/T) showing activation energy trends for (**A**) VCO vs. polymer, (**B**) VCO vs. polymer + Ag-ZnO composite, (**C**) VCO vs. polymer + Ag_2_O and (**D**) VCO vs. polymer + ZnO composite.

The Arrhenius-type plots of ln viscosity (cP) versus 1/T reveal distinct modifications in Ea when crude oil is treated with the bio-derived polymer (*S. macrophylla*) and its nanocomposite blends (Figure 8). Virgin crude oil (VCO) exhibited an activation energy of 213.6 kJ·mol^−1^, serving as the baseline for comparison. Incorporation of the polymer alone reduced Ea to 164.2 kJ·mol^−1^, corresponding to a 49.4 kJ·mol^−1^ reduction, while polymer–Ag_2_O and polymer–Ag-ZnO composites yielded reductions of 45.9 kJ·mol^−1^ and 42.3 kJ·mol^−1^, respectively. Interestingly, polymer–ZnO increased the activation energy to 218.3 kJ·mol^−1^, suggesting a counterproductive effect.

The observed reductions in activation energy indicate that the bio-derived polymer and its Ag-based nanocomposites facilitate molecular mobility and lower the energy barrier for crude oil flow. This aligns with the hypothesis that such additives disrupt wax–wax and wax–oil interactions, thereby mitigating buildup. The polymer alone demonstrated the most pronounced efficacy, highlighting the intrinsic potential of *S. macrophylla* as a sustainable wax inhibitor (Figure 8A). The Ag_2_O and Ag-ZnO polymer composites (Figure 8B,C), while slightly less effective, still provided substantial reductions, likely due to synergistic interactions between the polymer matrix and the metallic nanoparticles that enhance dispersion and adsorption at wax crystal surfaces.

Conversely, the ZnO polymer composite (Figure 8D) increased the activation energy, implying that ZnO may reinforce intermolecular associations or introduce steric hindrance that impedes flow. This finding underscores the importance of nanoparticle selection, as not all metal oxides confer beneficial effects in crude oil systems.

The observed reduction in Ea is primarily attributed to two factors: the adsorption of bio-derived polymers, which alters wax crystal nucleation and growth, and the synergistic effect of Ag_2_O and Ag-ZnO nanocomposites, which enhances the polymer’s ability to disperse crude oil components and reduce wax aggregation. However, the presence of ZnO presents an anomaly, as it increases Ea, suggesting its surface chemistry promotes wax–wax interactions and counteracts the polymer’s effect. These findings confirm that bio-derived *S. macrophylla* polymers are promising eco-friendly alternatives, with Ag-nanocomposites further boosting their efficacy, while ZnO is unsuitable. This mechanistic understanding of Ea is crucial for the rational design of tailored, sustainable, and high-performance flow assurance inhibitors [42].

**Figure 9:**
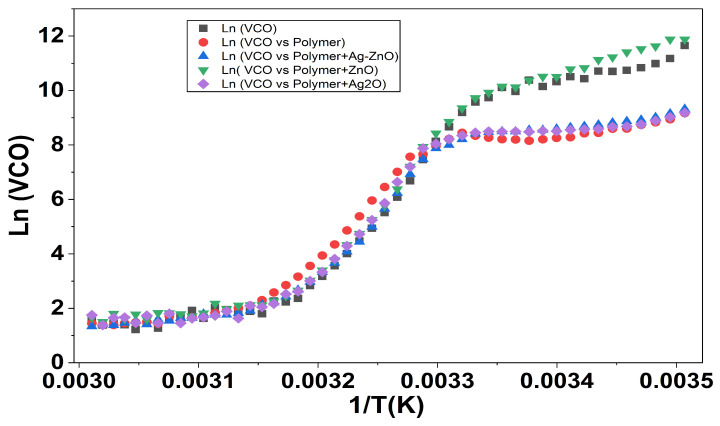
Combined comparison Arrhenius plots of Ln(VCO) versus reciprocal temperature (1/T).

The combined plot (Figure 9) demonstrates that bio-derived polymers and Ag-based nanocomposites consistently reduce the energy barrier for crude oil flow, while ZnO alone is counterproductive.

This comparative visualization strengthens the mechanistic argument that polymer adsorption and nanoparticle synergy can mitigate wax buildup, thereby improving flow assurance in crude oil systems [43].

Table 6 highlights the activation energies and the differential impact of *S. macrophylla* polymer and its nanocomposites on crude oil flow behavior. Virgin crude oil exhibited the highest baseline value (213.6 kJ·mol^−1^), while the polymer alone achieved the most pronounced reduction (−49.4 kJ·mol^−1^), underscoring its intrinsic inhibitory efficacy. Both Ag_2_O and Ag-ZnO composites also lowered the activation barrier (−45.9 and −42.3 kJ·mol^−1^, respectively), suggesting synergistic interactions between the biopolymer matrix and silver-based nanoparticles. In contrast, ZnO incorporation resulted in a slight increase (+4.7 kJ·mol^−1^), indicating that not all oxide nanocomposites are beneficial, and in this case, ZnO may reinforce wax–wax associations rather than disrupt them.

Hence, the table demonstrates that bio-derived polymers and Ag-based nanocomposites consistently reduce the energy required for crude oil flow, whereas ZnO alone appears counterproductive. This comparative evidence strengthens the case for selective nanoparticle integration in designing sustainable wax inhibitors.

### 3.6. CPM Analyses

**Figure 10:**
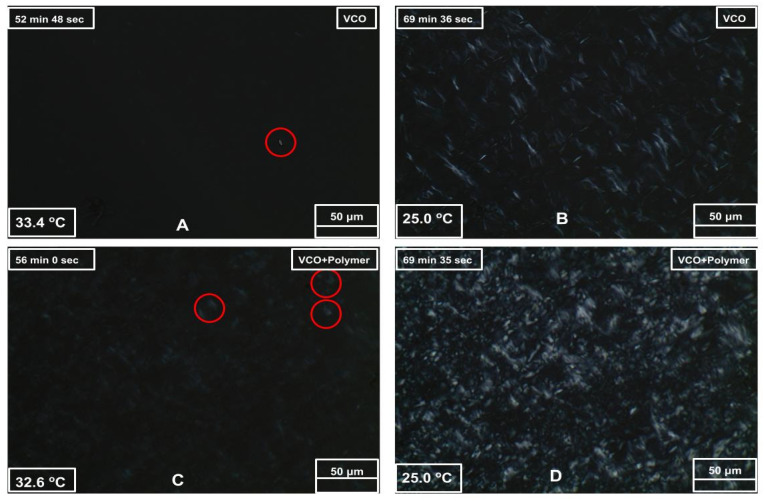

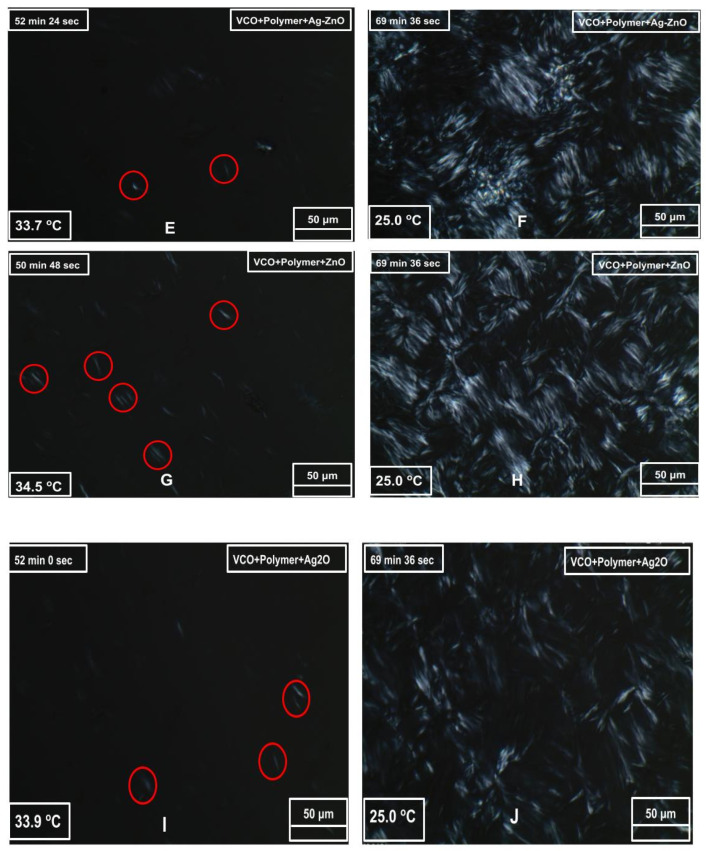
CPM micrographs at WAT for untreated crude oil and inhibitor-treated systems ((**A**) VCO at 33.4 °C; (**B**) VCO at 25.0 °C; (**C**) VCO + polymer at 32.6 °C; (**D**) VCO + polymer at 25.0 °C; (**E**) VCO + polymer + Ag-ZnO at 33.7 °C; (**F**) VCO + polymer + Ag-ZnO at 25.0 °C; (**G**) VCO + polymer + ZnO at 34.5 °C; (**H**) VCO + polymer + ZnO at 25.0 °C; (**I**) VCO + polymer + Ag_2_O at 33.9 °C; (**J**) VCO + polymer + Ag_2_O at 25.0 °C. The red circles denote the identification of wax crystals that emerge at the WAT.).

The CPM micrographs provide direct visual evidence of wax crystallization dynamics in virgin crude oil VCO and VCO treated with bio-derived polymer/metal nanocomposites. In the untreated VCO samples (Figure 10A,B), wax crystals begin to nucleate faintly at 33.7 °C, but as cooling progresses to 25 °C, the crystalline structures become elongated and densely packed. This progression reflects the natural tendency of paraffin molecules to self-assemble into ordered lattices once the system temperature falls below the wax appearance temperature (WAT).

By contrast, the VCO + polymer systems (C,D) show delayed and less pronounced wax nucleation. The initial faint crystals at ~32.6 °C are smaller and more dispersed compared to neat VCO, while at 25 °C, the crystalline network appears less compact. This suggests that the S. macrophylla-derived polymer interferes with the alignment of paraffin chains, reducing the rate and extent of crystal growth. More importantly, it has reduced the WAT by 0.8 °C. The mechanism is likely steric hindrance and adsorption: the polymer molecules adsorb onto nascent wax nuclei, disrupting their ability to grow into larger, interlocking structures. This inhibition translates into lower pour points and reduced deposition potential.

The polymer nanocomposite systems (Figure 10E–J) further highlight the synergistic role of metal nanoparticles, but they have elevated the WAT. In the Ag-doped ZnO and ZnO composites (Figure 10E–H), wax crystals appear at similar onset temperatures (~33–34 °C), but their morphology is markedly altered. The red-circled regions show smaller, irregularly shaped crystals, and at 25 °C, the crystalline texture is less continuous compared to neat VCO. This indicates that the nanoparticles act as heterogeneous nucleation sites, but their surface chemistry disrupts the regular lattice formation of paraffin molecules. Silver doping enhances this effect by modifying the electronic and surface properties of ZnO, increasing its affinity for polar components in crude oil and thereby interfering with wax aggregation.

Similarly, the polymer–Ag_2_O composite (Figure 10I,J) demonstrates a negative WAT effect, recording ~33.9 °C. Despite that, scattered crystals were captured at 25 °C, suggesting Ag_2_O nanoparticles, with their high surface reactivity, adsorb onto wax molecules and prevent them from forming extended crystalline domains. The mechanism here combines adsorption, steric blocking, and disruption of van der Waals interactions among paraffin chains.

Hence, the CPM images confirm that the bio-derived polymer alone reduces wax growth, but the incorporation of metal nanoparticles, despite a slight elevation of WAT, significantly enhances inhibition. The mechanism is twofold: (i) steric hindrance and adsorption by the polymer, and (ii) surface-mediated disruption of crystal growth by nanoparticles. This dual action delays WAT, reduces crystal size, and prevents the formation of dense wax networks. Such findings align with rheological and pour point data, underscoring the efficacy of *S. macrophylla*-based nanocomposites as sustainable, high-performance wax inhibitors [6,44].

### 3.7. Mechanistic Evaluation of Polymer–Nanoparticle Action

The quantitative morphometric analysis demonstrates that the addition of polymeric and polymer–nanoparticle inhibitors substantially alters the crystallization behavior of Dulang crude oil relative to the untreated sample. As summarized in Table 7, the untreated crude oil exhibited relatively few but large, interconnected birefringent crystals, characteristic of unrestricted crystal growth and extensive crystal–crystal association. Such needle-like structures readily interlock during cooling, producing a three-dimensional wax network capable of entrapping liquid hydrocarbons and consequently increasing viscosity, gel strength, and wax deposition tendency.

The polymer-only formulation significantly modified the wax crystal morphology compared with the untreated crude oil. The substantial increase in crystal population (782 crystals) accompanied by a moderate average crystal area (314.6 pixels) suggests that the polymer effectively disrupted the formation of continuous needle-like wax structures (see Figure 10). At the molecular level, this behavior can be attributed to the structural compatibility between the long hydrocarbon segments of the polymer and paraffinic wax molecules. During cooling, polymer chains adsorb onto the active growth faces of nascent wax crystals through intermolecular van der Waals interactions and partial co-crystallization with linear paraffin molecules. This adsorption interferes with the ordered packing of wax molecules, thereby suppressing anisotropic crystal growth and reducing the tendency for large, interconnected crystals to develop. Nevertheless, because the polymer provides limited additional nucleation sites, crystal formation remains primarily governed by homogeneous nucleation, resulting in only moderate control over crystal size and dispersion [45].

Among the investigated systems, the ZnO–polymer formulation produced 528 detected crystals with an average crystal area of 569.2 pixels. Although ZnO nanoparticles promoted heterogeneous nucleation, the relatively larger crystal size suggests that crystal growth and secondary aggregation were not completely suppressed. This observation indicates that ZnO primarily facilitates nucleation, whereas its ability to inhibit subsequent crystal growth is comparatively limited. In contrast, the Ag_2_O–polymer formulation exhibited the smallest average crystal area (256.2 pixels) together with a relatively low crystal population (175 crystals), indicating more effective suppression of crystal growth after nucleation. This behavior suggests that Ag_2_O nanoparticles, in conjunction with the polymer, enhance crystal dispersion by restricting crystal enlargement and minimizing crystal–crystal adhesion. At the molecular scale, oxygen-containing surface groups on Ag_2_O nanoparticles and functional groups within the polymer are proposed to generate steric and interfacial barriers around growing wax crystallites, thereby reducing their propensity to aggregate into a continuous network [46].

The Ag-ZnO + polymer nanocomposite demonstrated the most balanced crystallization behavior, producing 594 uniformly distributed crystals with an average crystal area of 491.1 pixels. The enhanced performance is attributed to the synergistic contribution of both nanoparticles. ZnO increases the density of heterogeneous nucleation sites, while Ag_2_O contributes to improved crystal dispersion and growth inhibition. Simultaneously, polymer chains adsorb onto the crystal surfaces through co-crystallization and intermolecular interactions with paraffinic hydrocarbons, forming a steric layer that restricts further crystal growth and aggregation. Instead of developing into large interlocking structures, the wax precipitates remain as fine, discrete particles suspended within the continuous oil phase. Such dispersed crystallites are considerably less effective in forming the interconnected three-dimensional wax network responsible for gelation and flow restriction [47].

**Figure 11:**
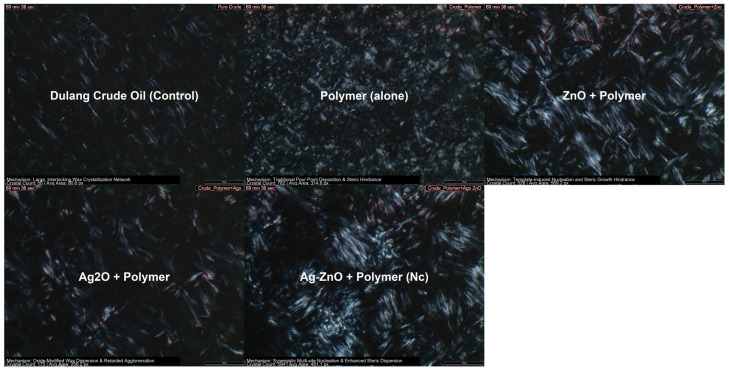
Comparative plate of annotated CPM micrographs showing target wax inhibitors (polymer and nanoparticles). The red boxes indicate the regions where birefringent wax crystals were computationally identified at the WAT, highlighting the nuclei and growth boundaries used for quantitative analysis.

The CPM findings align with the rheological analysis conducted through the automated Python-based pipeline and SciPy-supported morphometric quantification, confirming that inhibitor-treated samples exhibited reduced viscosity and enhanced flowability compared to untreated crude oil. The strong agreement between microscopic crystal morphology and macroscopic rheological behavior suggests that the polymer nanoparticle formulations primarily function by modifying the molecular organization of paraffin molecules during crystallization rather than dissolving the wax itself (Figure 11). Specifically, the nanoparticles alter nucleation kinetics by promoting heterogeneous nucleation, whereas the polymer inhibits crystal growth through adsorption-mediated co-crystallization and steric stabilization. The combined action disrupts the formation of large interconnected wax crystals, thereby mitigating wax deposition and improving the flow characteristics of the crude oil [48].

## 4. Conclusions

In this study, a bio-derived polymer from *S. macrophylla* (Mahogany) and its nanocomposites (ZnO, Ag_2_O, Ag-ZnO) were synthesized and evaluated for wax inhibition in Dulang crude oil. The results demonstrated measurable improvements in pour point depression (up to 5.17% reduction with polymer + ZnO), viscosity reduction (up to 91.6% with polymer + Ag-ZnO), and thermal stability (onset temperatures up to 262 °C with polymer + ZnO), all of which exceed the average pipeline operating temperature. The pure *S. macrophylla* polymer demonstrated significant rheology modification, reducing the activation energy (Ea) for flow by a considerable 49.4 kJ·mol^−1^ from the 213.6 kJ·mol^−1^ baseline, confirming its intrinsic potential to improve crude oil mobility. These findings confirm that the prepared nanocomposites enhance flow assurance under the tested conditions. The CPM image analysis demonstrated that the nanoparticles modify nucleation kinetics by inducing heterogeneous nucleation, while the polymer suppresses subsequent crystal growth through adsorption-mediated co-crystallization and steric stabilization. Unlike previously reported studies that focused on synthetic pour point depressants or edible biopolymers with food security concerns, this work introduces a non-edible, bio-derived polymer from *S. macrophylla* hybridized with metal oxide nanoparticles. To our knowledge, this is the first demonstration of a mahogany-derived polymer nanocomposite applied to waxy crude oil systems, integrating both rheological and thermal stability analyses. The combined evaluation of pour point depression, viscosity reduction, and thermogravimetric stability provides a more comprehensive assessment of wax inhibition performance than earlier single-parameter studies.

Despite demonstrating strong efficacy as a flow improver, this study also identified certain limitations. Future work is recommended to optimize the concentration of the polymer–nanocomposite formulations, extend the investigation to multiple crude oil systems to validate broader applicability, and incorporate advanced micro-characterization techniques (XRD, SEM, TEM) to confirm nanoparticle dispersion within the polymer matrix and provide direct evidence of nano-synergy.

## Figures and Tables

**Table 1:** Physical and chemical characteristics of Dulang crude oil.

Property	Dulang Crude Oil Value	Reference
Solid-to-Solid Crystalline (°C)	50	[17]
Cumulative Mol Percent (from C20 to C40)	25.86	[17]
Crystallization Temperature (°C)	55	[17]
Density (g/cm^3^)	0.837	[18]
Specific Gravity	0.8378	[18]
API Gravity	37.39	[18]
Specific Gravity	0.836	[26]
Molecular Weight (kg/kmol)	189.85	[26]
n-C5 (mol%)	0.004	[26]
C6 (mol%)	1.864	[26]
C7 (mol%)	7.713	[26]
C8 (mol%)	5.997	[26]
C9 (mol%)	3.675	[26]
C10 (mol%)	4.679	[26]
C11+ (mol%)	76.068	[26]
Wax Content (wt%)	19.5	This work
Viscosity at 25 °C (cP)	24,510.7	This work
Pour Point (°C)	33.25	This work
Wax Appearance Temperature (°C)	33.4	This work

**Table 2:** Major chemical compounds identified in *S. macrophylla* polymer using GC–MS.

Peak	RT (min)	Key Compounds Identified	Relative Area (%)	Notes
1	1.477–1.661	Pentane and n-Hexane isomers	-	Extraction solvent/diluent
2	34.701	Glycidyl palmitate, adipic acid esters	5.49%	Long-chain fatty acid esters
3	37.149	Glycidyl oleate, (Z)-octadecenals	59.61%	Unsaturated fatty acid esters, aldehydes
4	37.294	Succinic/adipic acid esters	9.37%	Complex ester derivatives
5	37.479	Glycidyl palmitate, myristoyl chloride	11.37%	Fatty acid derivatives
6	52.119	Dioxolane derivatives, substituted cyclohexenes	14.16%	High molecular weight oxygenated compounds

**Table 3:** Effect of *S. macrophylla* polymer and metal nanocomposites on the pour point of Dulang crude oil.

SN	Samples	Average Pour Point Temperature (°C)	Temperature Difference (°C)	Temperature Difference (%)
1	VCO (Dulang)	33.25	-	-
2	VCO + 2% Polymer	31.7	1.55	4.66%
3	VCO + 2% Polymer + 0.04% ZnO	31.53	1.72	5.17%
4	VCO + 2% Polymer + 0.04% Ag_2_O	32.75	0.5	1.5%
5	VCO + 2% Polymer + 0.04% Ag-ZnO	31.9	1.35	4.06%

**Table 4:** Comparative rheological performance of crude oil and inhibitor systems.

Shear Rate (s^−1^)	Virgin Crude (cP)	Polymer (cP)	Polymer + ZnO (cP)	Polymer + Ag_2_O (cP)	Polymer + Ag-ZnO (cP)
1	3.16 × 10^6^	1.8 × 10^6^	6.6 × 10^5^	2.54 × 10^6^	7.24 × 10^5^
10	7.30 × 10^4^	4.73 × 10^4^	3.703 × 10^3^	3.38 × 10^3^	3.39 × 10^3^
100	1.41 × 10^4^	3.61 × 10^3^	5.30 × 10^2^	4.77 × 10^2^	2.09 × 10^4^
200	1.10 × 10^4^	2.34 × 10^3^	3.05 × 10^2^	3.26 × 10^2^	9.12 × 10^2^

**Table 6:** Summary of the difference in activation energy of the inhibitors.

System	Slope (m)	Ea (J·mol^−1^)	Ea (kJ·mol^−1^)	Difference vs. VCO (kJ·mol^−1^)
Virgin Crude Oil (VCO)	25,690	213,586.7	213.6	—
VCO vs. Polymer	19,739	164,158.7	164.2	−49.4
VCO vs. Polymer + Ag-ZnO	20,608	171,342.6	171.3	−42.3
VCO vs. Polymer + ZnO	26,251	218,311.1	218.3	+4.7
VCO vs. Polymer + Ag_2_O	20,167	167,726.5	167.7	−45.9

**Table 7:** Quantitative morphometric analysis of wax crystals obtained from CPM images of untreated and inhibitor-treated Dulang crude oil at the wax appearance temperature.

Sample Name	Crystal Count	Avg Area (Pixels)	Wax Area %
Dulang Crude Oil (Control)	50	80.6	0.13%
Polymer (alone)	782	314.6	7.84%
ZnO + Polymer	528	569.2	9.58%
Ag_2_O + Polymer	175	256.2	1.43%
Ag-ZnO + Polymer (Nc)	594	491.1	9.30%

## Data Availability

The original contributions presented in this study are included in the article. Further inquiries can be directed to the corresponding authors.
